# *Rosmarinus officinalis* L. (rosemary) as therapeutic and prophylactic agent

**DOI:** 10.1186/s12929-019-0499-8

**Published:** 2019-01-09

**Authors:** Jonatas Rafael de Oliveira, Samira Esteves Afonso Camargo, Luciane Dias de Oliveira

**Affiliations:** 1 Departamento de Biociências e Diagnóstico Bucal, Instituto de Ciência e Tecnologia, Universidade Estadual Paulista (UNESP), Av. Engenheiro Francisco José Longo, 777 – Jardim São Dimas, São José dos Campos, SP CEP 12245-000 Brazil; 2 Department of Restorative Dental Sciences, University of Florida, College of Dentistry, Gainesville, FL 32610 USA

**Keywords:** *Rosmarinus officinalis* L., Rosemary, Biological activities, Phytotherapy, Therapeutic effects, Prophylactic effects

## Abstract

*Rosmarinus officinalis* L. (rosemary) is a medicinal plant native to the Mediterranean region and cultivated around the world. Besides the therapeutic purpose, it is commonly used as a condiment and food preservative. *R. officinalis* L. is constituted by bioactive molecules, the phytocompounds, responsible for implement several pharmacological activities, such as anti-inflammatory, antioxidant, antimicrobial, antiproliferative, antitumor and protective, inhibitory and attenuating activities. Thus, in vivo and in vitro studies were presented in this Review, approaching the therapeutic and prophylactic effects of *R. officinalis* L. on some physiological disorders caused by biochemical, chemical or biological agents. In this way, methodology, mechanisms, results, and conclusions were described. The main objective of this study was showing that plant products could be equivalent to the available medicines.

## Background

### Phytocompounds and pharmacological activities

*R. officinalis* L., popularly known as rosemary, is a plant belonging to the family Lamiaceae and originated from the Mediterranean region. However, it could be found all over the world. It is a perennial and aromatic plant, shrub-shaped with branches full of leaves, having a height of up to two meters and green leaves that exude a characteristic fragrance. *R. officinalis* may be used as a spice in cooking, as a natural preservative in the food industry, and as ornamental and medicinal plant [1–4].

**Fig. 1:**
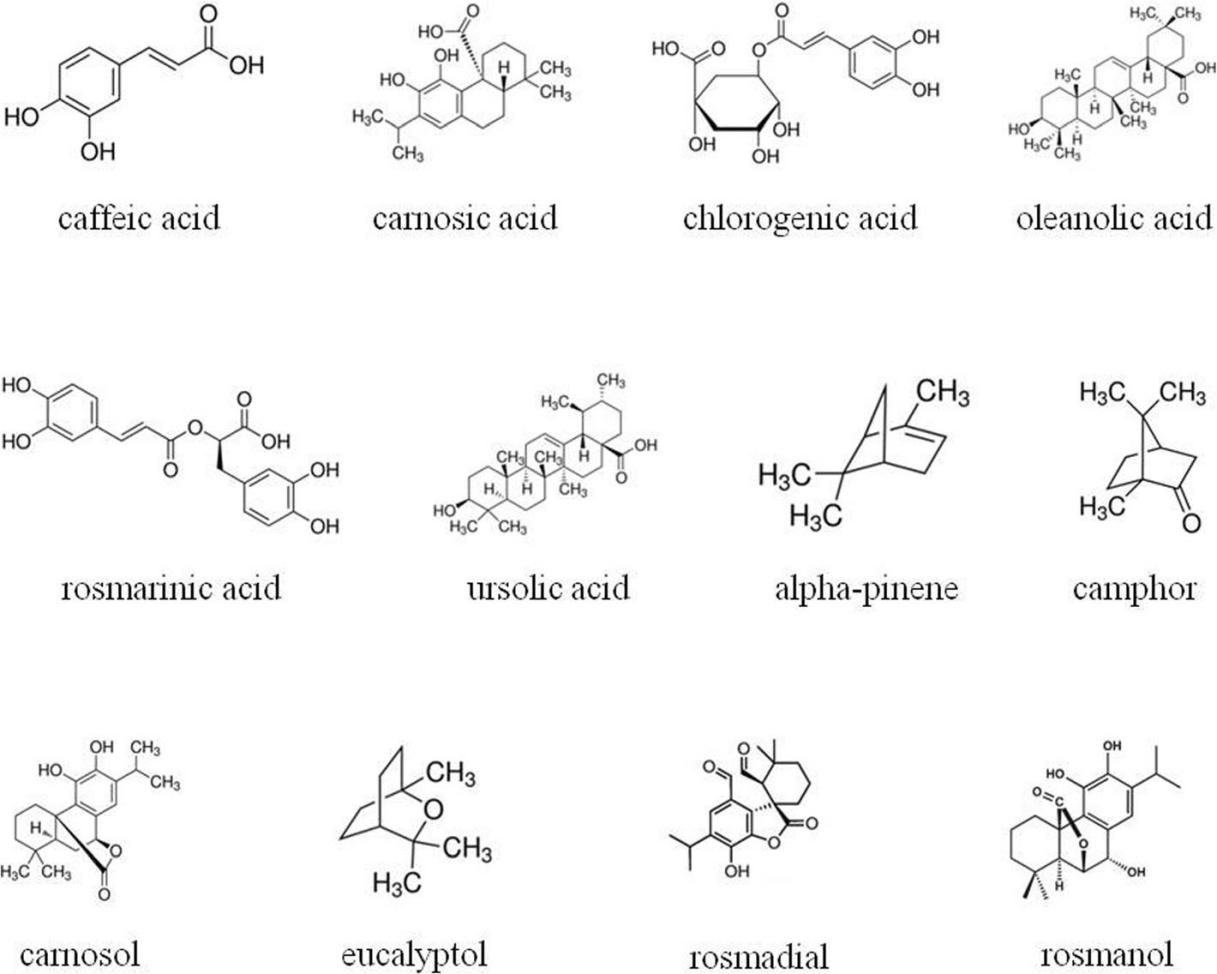
Phytocompounds present in *R. officinalis* L

Several phytocompounds presenting pharmacological activities may be isolated from essential oils and extracts of *R. officinalis* L. (Fig. 1), varying the concentration of these molecules in each specimen of the plant. The phytocompounds most reported include caffeic acid, carnosic acid, chlorogenic acid, monomeric acid, oleanolic acid, rosmarinic acid, ursolic acid, alpha-pinene, camphor, carnosol, eucalyptol, rosmadial, rosmanol, rosmaquinones A and B, secohinokio, and derivatives of eugenol and luteolin [5–8]. Pharmacological effects of phytocompounds from *R. officinalis* L. were showed in Table 1.

**Table 1:** Pharmacological effects of phytocompounds from *R. officinalis* L reported in the literature

Phytocompound	Pharmacological effect	Reference
Caffeic acid	a. Antibacterial	[168]
	b. Antioxidant	[169]
	c. Inhibitory effect of tumor cell immigration	[170]
	d. Inhibitory effect of tumor cell proliferation	[171]
	e. Protective effect of transplanted livers	[172]
	f. Apoptotic effect on tumor cells	[173]
Carnosic acid	a. Antiproliferative	[174]
	b. Protective effect of photoreceptor cells	[175]
	c. Antitumor	[176]
	d. Anti-inflammatory	[177]
	e. Inhibitory effect of digestive enzymes (lipase, α-amylase, and α-glucosidase)	[178]
	f. Suppressive effect of lipogenesis	[179]
Chlorogenic acid	a. Antioxidant	[180]
	b. Protective effect against lead-induced renal damage	[181]
	c. Protective effect against colitis	[182]
	d. Anti-infective	[183]
Oleanolic acid	a. Antiviral	[184]
	b. Protective effect against oxidative stress-induced apoptosis	[185]
	c. Antiproliferative	[186]
	d. Antitumor	[187]
	e. Antioxidant	[188]
Rosmarinic acid	a. Neuroprotective	[189]
	b. Protective effect against nicotine-induced atherosclerosis	[190]
	c. Complementary agent to the anticancer chemotherapy	[191]
	d. Anxiety control	[192]
	e. Antiproliferative	[193]
	f. Antiviral	[194]
Ursolic acid	a. Cytotoxic for tumor cells	[195]
	b. Anticancer	[196]
	c. Inducer of osteoblastic activity and reducer of osteoclastic activity	[197]
	d. Hypouricemic	[198]
	e. Proapoptotic	[199]
	f. Inductor of insulin sensitivity	[200]
	g. Protective effect against diabetic nephropathy	[201]
	h. Reducer of weight gain and atherosclerosis	[202]
Alpha-pinene	a. Antibacterial	[203]
	b. Antimicrobial	[204]
	c. Protective effect against aspirin-induced oxidative stress	[205]
	d. Protective effect against peptic ulcer	[206]
Camphor	a. Immunomodulatory	[207]
	b. Antiproliferative	[208]
	c. Hypoglycemic	[209]
	d. Antimicrobial	[210]
	e. Antifungal, antihyphal, and antibiofilm	[211]
Carnosol	a. Antiproliferative	[212]
	b. Protective effect against renal ischemia-reperfusion injury	[213]
	c. Antifungal	[214]
	d. Proapoptotic and proautophagic	[215]
	e. Anti-inflammatory	[216]
	f. Anti-atopic dermatitis	[217]
	g. Antidiabetic	[218]
Eucalyptol	a. Proapoptotic	[219]
	b. Antibiofilm	[220]
	c. Control of infection and inflammation	[221]
	d. Anti-inflammatory	[222]
	e. Antinociceptive	[223]
	f. Antiviral	[224]
Rosmanol	a. Antinociceptive, antidepressant, and anxiolytic	[82]
	b. Anticancer	[225]
Eugenol	a. Acaricidal	[226]
	b. Antifungal	[227]
	c. Chemotherapeutic on cervical cancer cells	[228]
	d. Antiproliferative	[229]
	e. Anti-inflammatory and antioxidative	[230]
Luteolin	a. Anti-inflammatory	[231]
	b. Anti-atopic dermatitis	[232]
	c. Proapoptotic and proautophagic	[233]
	d. Antimicrobial	[234]
	e. Antiproliferative	[235]
	f. Protection of microglia against rotenone-induced toxicity	[236]
	g. Inhibitory effect of glucocorticoid-induced osteoporosis	[237]

*R. officinalis* L. can promote several pharmacological effects due to the interaction between the molecules of the plant and the organic systems. The effects demonstrated by this plant include (1) ability to attenuate asthma, atherosclerosis, cataract, renal colic, hepatotoxicity, peptic ulcer, inflammatory diseases, ischemic heart disease [9, 10]; (2) antioxidant and anti-inflammatory actions of rosmarinic acid [11, 12]; (3) control of hypercholesterolemia and oxidative stress and relief of physical and mental fatigue [13]; (4) myocardial blood pressure reduction with rosmarinic acid [12]; (5) antiulcer action [14]; (6) lipid peroxidation reduction in heart and brain [15]; (7) antiangiogenic and neuroprotective effects of carnosic acid and carnosol [16]; (8) prevention of problems related to atherosclerosis [17]; (9) anticancer and antiproliferative effects [18–21]; (10) antiviral [22]; and antimicrobial actions [23]; (11) hepatoprotective [24], nephroprotective [25] and radioprotective-antimutagenic capacities [26]; (12) glycemia reduction [27]; (13) muscle relaxant and treatment for cutaneous allergy [28]; (14) ability to treat depressive behavior [29].

### Extraction methods

The extract of plant can be obtained from roots, stems, leaves, flowers, fruits, seeds, and bark. Therefore, fresh or dried samples can be used. However, according to Vongsak et al. [30], a higher level of flavonoids was detected in dried samples of *Moringa oleifera* leaves, as compared to fresh samples.

Drying techniques include [31]:

- *Air-drying*: a slower drying that can be performed in a range of days, weeks and even months. The process is conducted at room temperature exposing the plant to the atmospheric air. In this way, those unstable chemical compounds to the heat are not damaged.
- *Microwave-drying:* the drying time is faster than in the air-drying process due to the electromagnetic radiation. This process promotes collisions between the molecules of the plant, resulting in heating that causes water evaporation from the plant. Thus, many phytocompounds can be denatured and lose their pharmacological effectiveness.
- *Oven-drying:* the drying time is also fast by using heat to cause the water evaporation from the plant. Unlike microwave-drying, in this process, the phytochemicals are better preserved.
- *Freeze-drying:* a drying performed using sublimation method. The sample is initially frozen (− 80 °C) for 12 h and immediately lyophilized. This method favors the preservation of phytocompounds viability, obtaining higher levels of these molecules than in other drying methods.

Another relevant aspect is the size of the particles that can interfere in the extraction process. Since, the smaller the particle size, the higher the interaction between the plant sample and the solvent to obtain the extract. Thus, powder samples have better contact with the solvent than crushed samples. Nanoparticles containing *Centella asiatica* presented higher yields than microparticles, when in contact with methanol [32].

During the extraction, the active part of the plant, which contains the functional particles, is obtained, as well as the residual part. The raw extracts are composed of numerous active molecules, such as alkaloids, phenolic compounds, flavonoids, glycosides, and terpenoids. From this initial extract, other types can be obtained by various extraction methods, as can be observed in Table 2 [31].

**Table 2:** Extraction methods [31]

Method	Description
Maceration	Powdered or crushed materials are left in solvents for at least three days at room temperature under agitation. Them, the solution is filtered. Phytocompounds are released by breaking the cell wall of plant cells.
Infusion	The same maceration process is used, but the period is shorter, and the sample is boiled in specific volumes of water.
Decoction	The same maceration and infusion processes are used, but the extractions of thermostable compounds and substances from hard parts of the plant such as roots and bark are possible.
Percolation	The same maceration and infusion processes are used. The sample is placed in contact with boiling water, and the extraction is performed for about two hours. In the end, a concentrated extract is obtained.
Soxhlet extraction	The extraction process is performed in the Soxhlet extractor. Sample and solvent are placed in the apparatus. Upon heating the solvent, the solid particles from the substance are extracted. The generated liquid is absorbed and filtered. A more concentrated sample is obtained, and the heating of the solvent does not harm the compound.
Microwave assisted extraction	Use of microwaves to reach the molecules in a sample inside the solvent. The heating generated on the surface of the sample promotes changes in the structures of the chemical elements and favors the entry of the solvent into the material and consequently the extraction of the compounds.
Ultrasound-assisted extraction	Ultrasound (20 to 2000 kHz) is used for the extraction of the compounds. In this process, there is an increase in solvent contact with the sample, due to increased permeability of the plant cell wall. Sound waves impair the molecular integrity of the cell wall and thus favor the release of phytochemical agents.
Accelerated solvent extraction	In an automated way, compounds are extracted from solid and semi-solid samples, using small volumes of solvents, at high temperatures and pressures.
Supercritical fluid extraction	This extraction is performed using supercritical fluids as solvents, both in solid and liquid samples. Carbon dioxide (CO_2_) is the most commonly used fluid. This method can also be used for analytical purposes and the removal of unwanted substances or separation of a particular phytocompound in a sample. Temperature and pressure should be considered (~ 31 °C and 74 bar).

The solvent to extract active compounds may interfere with the final yield of these molecules. In *Psidium guajava* L. leaves extracts, the concentration of alkaloids, carbohydrates, flavonoids, saponins, and tannins was higher in ethanolic and hydroalcoholic solvents than petroleum ether, chloroform or water [33]. The presence or absence of some chemical constituents in the solvent can interfere in the pharmacological activities; e.g., the antioxidant activity was more prominent in *Garcinia atroviridis* methanolic extract than in aqueous extract. On the other hand, the aqueous extract of this plant presented better antihyperlipidemic effect [34].

The chosen method for extraction of compounds may interfere the yield of the sample. The use of high temperatures (90 °C) for *C. asiatica* extraction provided an increased yield of phenolic compounds which provided better antioxidant activity [35]. However, in microwave assisted extraction, a duplicate increase in the yield of *C. asiatica* triterpene was detected, compared to the Soxhlet extraction [36].

### The purpose of the study

Because of the immense variety of beneficial effects that *R. officinalis* L. has demonstrated, in vivo and in vitro studies were addressed in this Review (Table 3). Therapeutic and prophylactic effects of this plant on some physiological disorders caused by biochemical, chemical or biological agents were considered. This Review consisted of methodology, mechanisms, results, and conclusions of these studies. The main purpose of this work was demonstrating the ability of a medicinal plant (*R. officinalis* L.) to treat health problems, and showing its equivalence to any other medicine, concerning its beneficial effects.

**Table 3:** Pharmacological effects of *R. officinalis* L. summarized in this Review

Pharmacological effect	Product from *R. officinalis* L.	Main findings	Reference
Cardiac remodeling after myocardial infarction	Supplementation with leaves	a. Attenuation of cardiac remodeling b. Improvement of metabolism and reduction of oxidative stress	[47]
Control of body weight and dyslipidemia	Aqueous extract	a. Inhibition of the body weight gain b. Scavenging of free radical c. Inhibition of gentamicin (GM)-induced hepatotoxicity d. Antioxidant action e. DNA-protective effect	[60]
Neuroprotective effect on cerebral ischemia	Hydro-alcoholic extract	a. Absence of dyslipidemia effect b. Reduction of acute ischemic stroke lesion	[71]
Antinociceptive effect	Ethanolic extract	Pain control	[77]
Mono- and polymicrobial biofilms reduction	Glycolic extract	a. Antimicrobial effect b. Action on monomicrobial biofilms of *C. albicans*, *S. aureus*, *E. faecalis*, *S. mutans*, and *P. aeruginosa* b. Action on polymicrobial biofilms formed by *C. albicans* with each bacterium	[91]
Hepato-nephrotoxicity inhibition of the lead	Ethanolic extract	a. Protection of structure and function of liver and kidney against lead b. Stabilization of antioxidant proteins	[123]
Stress relief in situation of real danger	Hydro-alcoholic extract	a. Anxiolytic effect b. Stress control	[132]
Human tumor cells proliferation inhibition	Glycolic extract	Breast adenocarcinoma (MCF-7) and cervical adenocarcinoma (HeLa)	[91]
	Methanolic and ethyl acetate extracts	Epithelial colorectal adenocarcinoma (CaCo-2) and histiocytic lymphoma cell line (U-937)	[152]
	Aqueous extract	Esophageal squamous cell carcinoma (KYSE30) and gastric adenocarcinoma (AGS)	[153]
	Methanolic extract	Lung carcinoma (A549)	[154]

## Cardiac remodeling after myocardial infarction

### Background

Myocardial infarction is a condition characterized by the cardiac muscle necrosis, due to cell death caused by inflammation, which may be initiated by oxidative stress that produces cytokines synthesis, such as tumor necrosis factor-α (TNF-α) and interleukins (IL-1β and − 6); reabsorption of necrotic tissue; exacerbated collagen deposition; and hypertrophy. Both reactive oxygen species (ROS) and cytokines may induce the action of metalloproteinases (MMP), as well as the collagen accumulation, which is responsible for causing changes in the size, weight and function of the heart. Besides, the continued presence of metabolites from affected cells may also provide these changes. Thus, a forced adaptation of the organ to a new reality may occur, providing a cardiac remodeling that could lead to heart failure [37, 38]. In this manner, the use of antioxidants has been evaluated in these cases. Also, other types of medications have been used, such as the vasodilators prazosin, diltiazem, and felodipine, showing no satisfactory outcome regarding mortality reduction or hospitalization [39–41]. Positive inotropic drugs, which have a good hemodynamic effect, can also present significant side effects, about patient survival due to the neurohormonal activation and ventricular arrhythmias. Milrinone, flosequinan, pimobendan, ibopamine, and vesnarinone have caused an increase in mortality in chronic heart failure [42–45]. To control the oxidizing agents, the effectiveness of some natural products, including *R. officinalis* L., has been investigated, mainly by the presence of bioactive molecules with antioxidant capacities, such as rosmarinic acid, carnosic acid, and carnosol [46].

### Methodology

The effect of the supplementation with *R. officinalis* L*.* leaves was evaluated on cardiac remodeling after myocardial infarction in male Wistar rats [47]. For this, healthy animals and infarcted animals were fed with standard chow or chow containing 0.02% or 0.2% of *R. officinalis* L. leaves for 90 days. The animals were evaluated by transthoracic echocardiographic exam. Other analyses were performed on the left ventricle from the sacrificed animals, such as: (i) checking of the infarct extension in size and length; (ii) muscular viability by endocardial and epicardial circumferences; (iii) chemical mediators levels, such as TNF-α, IFN-γ, IL-10, MMP-2 and TIMP-1; (iv) total protein and lipid hydroperoxide levels; (v) enzymatic activity of glutathione peroxidase, superoxide dismutase, catalase; and (vi) cardiac metabolism checked by the activity of β-hydroxyacyl coenzyme-A dehydrogenase, lactate dehydrogenase, citrate synthase, Complex I (NADH:ubiquinone oxidoreductase), Complex II (succinate dehydrogenase), and ATP synthase.

### Findings

No deaths were observed in healthy animals; however, among infarcted animals, two deaths were verified in the groups that received standard chow and supplementation with 0.02% of *R. officinalis* L. leaves, besides one death in the group supplemented with 0.2%. Nevertheless, the infarction size was similar among infarcted animals and was not found any difference between the weight gain and systolic arterial pressure in all groups. The infarction generated an adverse cardiac remodeling, demonstrated by increased of left ventricular diameter; high collagen percentage; alterations in the diastolic and systolic functions; intensification of oxidative stress; metabolic changes evidenced by modification of enzymatic activity; increased of MMP-2 activity and decreased of IL-10, TNF-α, IFN-γ levels. However, the supplementation with *R. officinalis* L. leaves improved diastolic function, reduced muscle hypertrophy, provided morphological and functional changes in the heart of infarcted animals, verified by increased β-oxidation of fatty acids and reduced lactate oxidation, besides improved respiratory chain performance. *R. officinalis* L. significantly decreased oxidative stress, even though the used concentrations provided different scenarios regarding the diastolic function and hypertrophy, since the supplementation with 0.02% presented lower left atrium and supplementation with 0.2% demonstrated higher Complex II activity. Additionally, collagen percentage, cytokines levels, and MMP-2 activity were not altered with any of the supplementations.

#### Action mechanisms

Molecular and cellular changes in the heart are responsible for the clinical problems. Thus, metabolic pathways and antioxidants could be the interaction forms of *R. officinalis* L. with living tissue [47, 48].

Metabolic changes, oxidative stress, and redox signaling are factors that contribute to cardiac remodeling [49]. Murino Rafacho et al. [47] demonstrated that animals submitted to myocardial infarction and supplemented with *R. officinalis* L. leaves showed a higher fatty acids oxidation and respiratory chain improvement, similar to the metabolism of non-infarcted animals. Besides, they found a decrease of oxidative stress and enzymatic activity in cardiac tissue, using supplementation.

Oxidative stress, caused by the action of reactive oxygen species (ROS), can be controlled by antioxidant enzymes such as superoxide dismutase and catalase [50]. The enzyme superoxide dismutase is the first to protect the mitochondria against harmful effects of ROS during cardiac remodeling [51]. According to Chohan et al. [52], *R. officinalis* L. can function as an antioxidant enzyme and remove superoxide radicals from the tissue.

Nuclear factor (erythroid-derived 2)-like 2 (Nrf2) is responsible for the transcription of genes encoding antioxidant enzymes, and an increase in its expression has been noticed after treatment with *R. officinalis* L. [53, 54]. Therefore, supplementation with this plant has demonstrated an antioxidant characteristic comparable to its healthy cells [47].

## Control of body weight and dyslipidemia

### Background

Lipid metabolism may be altered and lead to increased levels of total cholesterol, low-density lipoprotein cholesterol and triacylglycerols, in the blood, causing cardio- and cerebrovascular disorders [55]. The use of some medications may induce to dyslipidemia, including antirheumatics [56], second-generation antipsychotics [57], antiretrovirals [58], and antibiotics (gentamicin) [59]. In this way, plant products to control dyslipidemia has been investigated.

### Methodology

The toxicity caused by gentamicin was attenuated in Sprague-Dawley rats with administration of *R. officinalis* L. extract [60]. In this study, the animals received gentamicin by intraperitoneal injection and 8% *R. officinalis* L. aqueous extract orally (10 mL/kg), the control groups were treated with saline solution (0.9% NaCl) or gentamicin (60 mg/kg). Doses were daily given over 10 days.

### Findings

The body weight of the animals increased significantly in the group treated with gentamicin compared to the control group (saline), demonstrating that the antibiotic could change the body mass. On the other hand, there was an inhibition of the body weight gain using the co-administration of the extract. Besides, the plant product also significantly reduced the liver weight of the animals compared to the group treated with antibiotic. The liver injuries caused by gentamicin were reversed with the administration of the extract. The harmful effects of this antibiotic were attenuated with plant extract at the liver level, providing a significant decrease in alanine aminotransferase and aspartate aminotransferase activity and total bilirubin levels. *R. officinalis* L. extract presented hypolipidemic effect, as evidenced by significant reductions in total cholesterol, phospholipids, triacylglycerols and atherogenic index. Additionally, the co-administration of the extract also protected against DNA damage, demonstrated by the absence of genetic material fragmentation in treated animals.

#### Action mechanisms

Plants from Lamiacea family are rich in phytocompounds, such as catechins, coumarins, and cinnamic acid. These molecules are responsible for exerting significant antioxidant activity, as well as quercitin, luteolin, kaempferol, and rosmarinic, hydrocafeic and caffeic acids [61]. Thus, *R. officinalis* L. can protect the organism against hyperlipidemic and hepatotoxic effects promoted by some products, as gentamicin [60]. This antibiotic can affect the liver and enhance the enzymatic activity of aspartate transaminase (AST) and alanine transaminase (ALT), as well as increase the bilirubin level and decrease the protein synthesis. Also, gentamicin is responsible for increasing the levels of triglyceride, cholesterol, and phospholipid, besides improving the pancreatic lipase activity [60, 62]. Hyperlipidemia can favor the emergence of heart disease and contribute for an increase of body weight.

*R. officinalis* L. acts decreasing the hydrogen peroxide levels, which promotes protection against oxidative stress caused by a toxicity inducer, as gentamicin. In fact, the plant can reduce the ROS production and protect the hepatic tissue from damage in DNA, proteins, and membranes [60]. Additionally, *R. officinalis* L. can increase the activity of phase I and II enzymes, providing a detoxification effect [63].

Regarding the dyslipidemia aspects, *R. officinalis* L. inhibits the activity of 3-hydroxy-3-methylglutaryl coenzyme A(HMG-CO) reductase. This provides a significant cholesterol reduction by oxidative stress [60]. Yokozawa et al. [64] reported that polyphenols can induce fecal excretion of total cholesterol and bile acids. Thereby, a decreased level of cholesterol in plasma can be observed because it is used in the biliary juice synthesis. Besides, an absorption reduction of this lipid in the intestine also is verified due to the low disposition of bile acids.

## Neuroprotective effect on cerebral ischemia

### Background

The localized blood flow reduction in the brain is known as cerebral ischemia, caused by arteries obstruction or systematic hyperfusion, causing irreversible damage. Inflammation and oxidative stress may be related to this physiological disorder [65]. The unexpected decrease of vital supplies (oxygen and nutrients), due to ischemia, may lead to stroke [66], which is caused by the edema from the rupture of the blood-brain barrier [67]. This liquid accumulation contributes to increase the brain mass [68] and consequently promote cell death [69]. Thus, recombinant tissue plasminogen activator (TPA) has been used for the treatment of this problem; however, this product has caused a worsening of the lesions as a result of the cerebral ischemia, contributing to the increase in the infarct size, cerebral edema, and hemorrhage intracranial [70]. Thus, alternative products for the treatment has been studied, including the plant products.

### Methodology

Hydroethanolic extract obtained from the *R. officinalis* L. leaves has been demonstrated in providing brain tolerance to artificially induced ischemia [71]. For this analysis, the authors promoted occlusion of the middle cerebral artery with intraluminal nylon filament implantation for 60 min in adult male Wistar rats, restoring blood flow after this period. Moreover, the animals were previously treated with the extract at 50, 75 or 100 mg/kg/day or with the vehicle (control) for 30 days. Ischemia induction occurred 2 hours before the last treatment. Non-ischemic animals were also included in the study for comparative purposes. The reperfusion period was 24 h and then the analyses were performed on the animals, including: (i) total cholesterol (TC), triglyceride (TG), low density lipoprotein (LDL-c) and high density lipoprotein (HDL-c) levels, quantified on the 30th day of treatment before surgery; (ii) neurological functions assessed by means of scores, such as 0 (“*no neurological dysfunction*”), 1 (“*failure to extend opposite forepaw*”), 2 (“*circling to the contralateral side, when held by tail with feet on floor*”), 3 (“*falling to the left*”), 4 (“*unable to bear weight on affected side*”/“*no spontaneous walking and a depressed level of consciousness*”), and 5 (“*death*”); and (iii) neurological behavior, including volume of the infarct and edema and permeability of the blood-brain barrier.

### Findings

After the 30th day, all animals gained weight; however, it was lower in the group treated with 100 mg/kg. *R. officinalis* L. extract decreased TC, TG, and LDL-c and increased HDL-c levels. In the treated groups were observed low levels of LDL/HDL and TG/HDL after 30 days. These data demonstrated that *R. officinalis* L. extract had no dyslipidemic effect. Regarding the neurological functions, the plant extract contributed to reducing the neurological deficit, since the untreated group presented score 3 (*“falling to the left”*), and after using doses of 50, 100 and 75 mg/kg the score was 1 (*“failure to extend opposite forepaw”*). The extract also provided a reduction in the infarction volume, presenting an excellent protection in the groups treated with 75 and 100 mg/kg. On the other hand, in untreated groups, the induced ischemia caused severe infarction in the subcortex and cerebral cortex regions. The edema formation was controlled in the animals treated with *R. officinalis* L. extract since protection against rupture of the blood-brain barrier and non-extravasation of liquid were observed.

#### Action mechanisms

Seyedemadi et al. [71] found that *R. officinalis* L. hydroethanolic prevented the rupture of the blood-brain barrier, as well as the cerebral edema, infarction, and neurological problems, in a murine model with middle cerebral artery occlusion. This can occur due to the ability of *R. officinalis* L. to prevent the mitogen-activated protein kinase (MAPK) phosphorylation, which provides the blockade of nuclear factor kappa B (NF-kB) activation. This blocking will decrease the expression of nitric oxide synthase (iNOS) and cyclooxygenase-2 (COX-2). During the inflammatory process, leukocyte activity and action of proinflammatory enzymes and other mediators, such as nitric oxide (NO), interleukin 1 beta (IL-1β), and tumor necrosis factor-alpha (TNF-α), can significantly decrease [72]. In ischemia pathogenesis, the oxidative stress is remarkable and can lead to the rupture of the blood-brain barrier and neurons death [73]. According to Huang et al. [74], *R. officinalis* L. can promote reduction of lipid peroxidation, hydroxyl radical, and hydrogen peroxide action in some tissues, such as cerebral, renal, cardiac, and serum. This fact shows that the plant can control the release of oxidative stress promoting molecules which are harmful to brain health.

## Antinociceptive effect

### Background

Cyclooxygenase inhibitor medicines have been used to treat pain, such as non-steroidal anti-inflammatory drugs (NSAIDs), although the prolonged use of these medications may lead to cardiovascular, renal, and gastric complications [75, 76]. In contrast, products obtained from medicinal plants can operate synergistically with these medicines to control pain.

### Methodology

The synergistic and antinociceptive activities of *R. officinalis* L. ethanol extract was reported in a study conducted in Wistar rats [77]. The animals were previously treated with plant extract, phytocompounds (ursolic acid and oleanolic acid), ketorolac, an NSAID, and ketorolac associated with plant products. Nociception was induced by subcutaneous injection of 1% formalin in the right paw dorsum.

### Findings

Plant extract (0.58 μg/paw) and ketorolac (0.88 μg/paw) provided antinociception of 66.5%. Higher doses of these products (10 μg) showed values of 38.5 and 42.6%, respectively. Thus, the drug interaction presented more effective antinociceptive action using lower doses. The administration of ursolic acid and oleanolic acid provided antinociception of 48.7 and 47.5%, respectively. Additionally, the association of extract or ursolic acid with ketorolac presented a nociception reduction of 61.1 and 71%, respectively. The phytocompound may be one of the responsible for the synergistic and antinociceptive effects of *R. officinalis* extract.

#### Action mechanisms

Antinociceptive activity can be increased with synergism between NSAIDs and plant products, such as extracts and phytocompounds from *R. officinalis* L. Thus, doses of analgesics could be reduced, as demonstrated by Beltrán-Villalobos et al. [77] which treated rats with ketorolac associated with *R. officinalis* L.

*R. officinalis* L. has caused inhibition of pain, according to preclinical studies, due to its interaction with opioid and 5-hydroxytryptamine (5-HT1A) receptors [78–80]. In inflammation model, *R. officinalis* L. essential oil showed effective antinociceptive activity in association with endogenous opioids in the serothogenic system, via 5-HT 1A receptor [79].

Lee et al., [81] found that eugenol, a phytocompound from *R. officinalis* L., can act on γ-aminobutyric acid type A (GABAA) receptor modulation in trigeminal ganglion neurons. Other compounds of this plant, such as rosmanol, cirsimaritin, and salvigenin, have also shown antinociceptive effect, by GABAA receptor modulation [82]. Heperidine, obtained from *R. officinalis* L., has also been induced inhibition of pain by interacting with transient receptor potential cation channel subfamily V member 1 (TrpV1) [83]. These authors also found that the interaction of hyperidin with ketorolac has shown a synergistic antinociceptive effect on inflammatory pain. Other phytocompounds from *R. officinalis* L. such as α-phellandrene and ursolic acid can also act on TrpV1 receptors [84, 85]. The antinociceptive effect of ursolic acid is modulated by cyclic guanosine monophosphate (cGMP) and 5-HT_1A_ [85]. Poeckel et al. [86] found that phytocompounds from *R. officinalis* L. can decrease ROS formation, inhibiting 5-lipoxygenase, COX-2, and leukocytes, and blocking Ca^2+^ channels in polymorphonuclear cells.

## Mono- and polymicrobial biofilms reduction

### Background

Biofilms are formed by microbial communities of different species adhered to the biotic or abiotic substrate, being surrounded by polysaccharide extracellular matrix produced by the microorganisms. This structure offers protection to the microorganisms against the external environment, actions of the host’s defense system and antimicrobial agents [87, 88]. The proportion of microbial cells and extracellular matrix may range between 10 and 25% of cells and 75–90% of polymeric substances [89]. The microorganism arrangement in these three-dimensional structures gives them about a thousand times more antimicrobial resistance than in planktonic cells, being directly related to cases of infectious diseases [90]. Therefore, the development of new products or strategies to combat microorganisms in biofilms is important. Another concern of the scientific community is the constant emergence of antimicrobial-resistant strains, which has been stimulating the search for alternative methods to control pathogenic microorganisms.

### Methodology

Phytotherapy is a wide field that can use plant products as an antimicrobial. The results of some studies have been increasingly promising and motivating. *R. officinalis* L. glycolic extract is an example of this, since its ability to control mono- and polymicrobial biofilms were cited [91]. In this study, the authors proposed to evaluate the effect of this plant extract on microorganisms that cause oral infections, such as *Candida albicans*, responsible for pseudomembranous/erythematous candidiasis and angular cheilitis [92]; *Staphylococcus aureus*, related to periodontitis due to its presence in supra- and subgingival biofilms [93]; *Enterococcus faecalis*, associated with asymptomatic endodontic infections characterized by formation of periapical lesions [94]; *Streptococcus mutans*, one of the agents that promote the development of dental caries [95]; and *Pseudomonas aeruginosa* linked to more aggressive periodontitis [96]. According to de Oliveira et al. [91], the action of *R. officinalis* L. extract was analyzed both on monomicrobial biofilms of each species and polymicrobial biofilms formed by *C. albicans* associated with *S. aureus*, *E. faecalis*, *S. mutans* or *P. aeruginosa*. These microbial associations were carried out once these species cause important clinical manifestations or present peculiar behavior when they are together. It has been reported that *C. albicans* may favor the development of *S. aureus* [87] and, besides, this bacterium was found in 27% of candidemia in nosocomial infections [97]. The association of *C. albicans* with *E. faecalis* both are benefited, and their pathogenicity may decrease, unlike when they are alone [98]. The interaction of *C. albicans* with *S. mutans* results in an extremely virulent biofilm on the teeth [99]. The development of *C. albicans* may be regulated by enzymes secreted by *P. aeruginosa*; these proteins affect the process of cellular respiration and hyphal formation [100]. Therefore, mono- and polymicrobial biofilms were formed in microplate for 48 h. After, planktonic cells were discarded by washes with saline solution (0.9% NaCl) and the biofilms were treated with *R. officinalis* L glycolic extract (200 mg/mL) for 5 min, considering its use as dentifrice or mouthwash. The affected cells were removed by other washes with saline solution, and the biofilms were disaggregated by ultrasonic homogenizer using a potency that caused no damage to the structure of the microorganisms (25%/30 s). Subsequently, the generated microbial suspension was diluted in saline solution and added in solid medium to form colonies. For polymicrobial biofilms, the suspensions were added in selective medium to determine how much each specie was affected in the mixed biofilm, both by the microbial interaction and by the extract action. This analysis was performed by counting of colony-forming units, being presented in concentration per milliliter (CFU/mL).

### Findings

*R. officinalis* L. extract provided a significant monomicrobial biofilms reduction after 5 min treatment, with rates of 99.96 ± 0.07% for *C. albicans*; 67.84 ± 12.05% for *S. aureus*; 77.64 ± 15.67% for *E. faecalis*; 79.32 ± 7.34% for *S. mutans*; and 98.23 ± 2.17% for *P. aeruginosa*. Regarding the polymicrobial biofilms, the plant extract was also effective due to a decreased CFU/mL concentration observed in the treated groups. In the association of *C. albicans* with *S. aureus*, the yeast was more affected (89 ± 13.89%) compared to the bacterium (56.75 ± 22.58%). In the biofilm of *C. albicans* with *S. mutans* was also observed reductions of 92.04 ± 5.24% and 64.55 ± 15.12%, respectively. On the other hand, the associations of *C. albicans* (85.87 ± 17.48%) with *E. faecalis* (93.03 ± 2.44%) and *C. albicans* (85.19 ± 10.48%) with *P. aeruginosa* (83.33 ± 17.79%) significant differences were not found. These results demonstrated the potential antibiofilm effect of *R. officinalis* L. extract on microorganisms that may cause oral infections, as well as the possibility of its insertion in oral hygiene materials to control biofilms adhered to surfaces, such as teeth, oral mucosal, prostheses, and orthodontic appliances.

#### Action mechanisms

Plant products have shown ability to act on biofilms adhered to a surface [91]. In this way, these products can inhibit the biofilm formation, prevent the planktonic cells adhesion, and, consequently, block the microbial colonization [101, 102].

Plant extracts and phytocompounds can also impair the microbial colonization. Microbes grown together with plant products have shown less adhesion capacity, resulting in a biofilm formed by adhered cells that can be easily removed [103].

A possible interaction target could be the bacterial lipid bilayer. Carvacrol and thymol are chemically attracted to the phospholipids of bacterial cytoplasmic membrane and this interaction promotes loss of membrane integrity and loss of cellular material, such as ions, adenosine triphosphate (ATP), and genetic material [104, 105]. The hydrophobicity presented by some phytocompounds favors their diffusion through the polysaccharidic matrix of the biofilm, promoting the destabilization of the microbial community [103].

Another proven mechanism is the interaction of plant products with adhesive proteins located on the microbial surface, preventing the attachment of new microorganisms to the substrate or weaken the attachment of adhered microorganisms [103].

In fungal species, da Silva Bomfim et al. [106] demonstrated that *R. officinalis* L. essential oil affected the size of *Fusarium verticillioides* microconidia, a fungus responsible for infecting grains such as corn and wheat. This morphological alteration can impair the development of the fungal biofilm. The mechanism involves turgor pressure reduction on the fungal cell wall, as well as changes in the cell surface caused by the need of osmotic equilibrium restoration [107].

The antifungal effect of *R. officinalis* L. is result of its interaction with the cell membrane and cell wall. The integrity of these structures is affected and all cytoplasmic material is dispensed in the medium. This fact can be verified by the presence of wrinkled cells in the fungal biofilm [106, 108].

Interruption of fungal cell growth, by the action of plant products, can be related to the ergosterol biosynthesis inhibition, which is present in the cell membrane, as it is occurred with antifungal drugs. In this sense, the membrane integrity is affected and the functionality of its proteins is also impaired, causing problems related to osmoregulatory process, cell growth, and fungal proliferation [109].

Additionally, the antifungal activity of *R. officinalis* L. essential oil has been related to the inhibition of *C. albicans* germ tube formation, an important virulence factor used for penetration and diffusion in organic tissues [110]. This effect occurs due to oxidative stress generated by the plant product, which triggers alterations in enzymatic activity and potential of mitochondrial cell membrane. Thus, it is possible inhibiting the germ-tube formation, yeast growth, and promoting the fungal death [111].

## Hepato-nephrotoxicity inhibition of the lead

### Background

Lead (Pb) is a toxic heavy metal and harmful to living things when mainly carried by food, water, and air. It may present accentuated toxicity to the liver and kidneys [112] as evidenced by a post-mortem analysis in individuals intoxicated by Pb [113]. The contact with Pb is initiated by its entry in the organism from drinking water contaminated with the metal from the pipelines, canned foods due to solder used in the cans, and ceramic enamels [114]. Lead may provide a variety of disorders, including hematological [115] and immunological changes [116], cardiac [117], nervous [118], metabolic and reproductive problems [119], and cancers [120]. However, ethylenediaminetetraacetic acid (EDTA) calcium disodium salt, magnesium dimercaptosuccinic acid (DMSA), D-penicillamine (PCA), and dimercaprol (BAL) have been used to treated Pb poisoning cases. These substances are chelating and provide Pb reduction in the body [121]. However, these medications can cause intoxication due to high dosage or allergic reaction regards to the penicillin. The common side effects are: (i - EDTA) nephrotoxicity, headache, fatigue, myalgia, thirst, fever, nausea and vomiting, sneezing, nasal congestion, lacrimation, rashes, anemia, and hypotension; (ii - DMSA) nausea, diarrhea, rashes, transient elevation of the serum aminotransferase; (iii - PCA) rheumatoid arthritis, urticaria, maculopapular reactions, lupus, pemphigoid, myasthenia gravis, renal toxicity progressing to nephrotic syndrome, leukopenia, thrombocytopenia, and aplastic anemia; and (iv - BAL) rise in the blood pressure, tachycardia, vomiting, abdominal pain, headache, burning sensation in mouth and throat, lacrimation, blepharospasm, rhinorrhea, sweating, anxiety, fever, hemolytic anemia [122].

### Methodology

Alternatively, *R. officinalis* L. ethanolic extract has been evaluated as a protective option against the hepato-nephrotoxic effect caused by the Pb [123]. In this study, male albino rabbits received distilled water (control group), *R. officinalis* L. extract or lead acetate (PbA) for 30 days at 30 mg/kg. Also, another group of animals received plant extract for 30 days and then PbA for the same period. Blood of sacrificed animals was collected for analysis of total erythrocyte, packed cell volume, hemoglobin, mean cell volume, mean corpuscular hemoglobin concentration, and total leukocyte, granulocyte, lymphocyte, and monocyte. A biochemical analysis was performed from the serum of the animals, verifying the presence of markers related to damage in the liver and kidneys, as well as activities of aspartate transaminase (AST), alanine aminotransferase (ALT), alkaline phosphatase (ALP), from liver, and levels of urea (ERU) and creatinine (CRE), from kidneys. The activity of catalase (CAT), superoxide dismutase (SOD), and malondialdehyde (MDA) were quantified. Besides, lipid peroxidation (LIP), glycogen (GLY), and tissues protein (TSP) levels were checked. Histopathological and histochemical analyses were performed by hematoxylin/eosin staining and mercury bromophenol blue method, respectively.

### Findings

Animals exposed to the PbA showed significantly reduce of activity and body weight. Absolute weight of liver and kidneys also decreased to 42 and 62%, respectively. On the other hand, the treatment with *R. officinalis* L. extract previously promoted normalization of the absolute weight of liver (66%) and kidneys (80%). These data indicated the protective effect of the plant extract against the damages caused by Pb, regarding the changes in the mass of the organs. The animals exposed for 30 days to PbA presented a decreased in total erythrocyte, packed cell volume, hemoglobin, mean cell volume and mean corpuscular hemoglobin concentration. The number of total leukocytes (neutrophils and monocytes) was increased; however, the levels of lymphocyte and eosinophil were dramatically decreased. In rabbits pretreated with plant extract, the cell concentration was similar to the control group, demonstrating that the *R. officinalis* L. extract had a protective effect, even with prolonged exposure to the Pb. The production of markers related to hepatic and renal damages was higher in animals exposed to the PbA. The activity of AST (173%), ALT (259%), ALP (162%) and the levels of ERU (161%) and CRE (153%) were significantly increased. However, in animals pretreated with extract, lower concentrations of AST (168%), ALT (129%), ALP (136%), ERU (121%) and CRE (112%) were observed. Thus, the potential of *R. officinalis* L. extract to protect the organisms against the harmful effects of the Pb was verified by biochemical tests. In animals exposed to PbA, the activity of antioxidant enzymes was significantly reduced, including CAT (52%) and SOD (47%), whereas MDA level (181%) was increased. In samples obtained from kidneys suspension, higher rates of CAT (57%), SOD (62%), and MDA (375%) were found. CAT (26%), SOD (45%) and MDA (63%) levels from the liver, and CAT (16%), SOD (33%) and MDA (87%) levels from kidneys were controlled only in animals pretreated with plant extract. A significant glycogen reduction was observed in the groups treated by PbA, both in the liver (41%) and in the kidneys (21%). In contrast, the animals treated with *R. officinalis* L. extract presented indexes of 20 and 7%, respectively. The tissue protein levels were statistically similar between the group exposed to PbA and pretreated with extract, presenting reductions in both cases. Histopathological analyses showed no alterations in the liver of rabbits treated with *R. officinalis* L. extract or distilled water (control), as well as in pretreated animals. Histopathological changes were observed instead in animals exposed to the PbA, demonstrating necrotic areas. Regarding the kidneys, abnormalities were not observed in the control, pretreated and treated groups. In contrast, severe tissue and pathological disorders were observed in groups exposed to the PbA. High concentrations of neutral mucopolysaccharides of hepatocytes and renal tubules were demonstrated by histochemical analysis in rabbits from control and treated groups. A carbohydrates reduction was observed in animals treated by the PbA, both in their liver and kidneys; however, a higher concentration was found in the animals preliminarily treated with *R. officinalis* L. extract. As for proteins, a reduction was observed in the exposed groups to the PbA, both in the liver and kidneys. Despite this, the protein content was not affected in the group pretreated with the plant extract. Hence, the protective effect of *R. officinalis* L. extract was also histologically proven against hepato-nephrotoxicity of the Pb.

#### Action mechanisms

Mohamed et al. [123] demonstrated the protective ability of *R. officinalis* L. ethanolic extract in PbA-induced hepato-nephrotoxicity. The study was carried out in rabbits and showed that PbA caused a significant hepatic and renal dysfunction, compared to the animals not contaminated with PbA. These dysfunctions can be results of changes in the cell membrane integrity, increased ROS production, and lipid peroxidation [124]. In the study by Mohamed et al. [123], rabbits pre-treated with *R. officinalis* L. were protected against the harmful effects of PbA. The plant extract provided a reduction of diffuse vacuolar cytoplasmic degeneration in hepatocytes and renal tubules, besides decreased infiltration of lymphocytes in the liver and kidneys.

Additionally, in this study, loss of glycogen and liver and renal proteins was identified after exposure to the PbA. This fact occurred due to the interference of PbA in absorption and metabolism of glucose, as well as in induction of protein catabolism [125]. On the other hand, *R. officinalis* L. inhibited the PbA action, and the animals were not metabolically affected.

Regarding the hematological aspects, anemia was diagnosed in animals poisoned by PbA, probably caused by enzymatic activity related to the metabolism of cell and metal [126]. Inflammation induced by the intoxication promoted elevated levels of leukocytes, neutrophils, and monocytes. However, treatment with *R. officinalis* L. provided low levels of anemia and normal levels of white blood cells.

The protective hepato-nephro effect of *R. officinalis* L. can be related to the interferences in oxidative stress and lipid peroxidation caused by exposure to the PbA. The plant extract restored the constitution of endogenous antioxidants that were lost by the PbA intoxication, besides regularizing the high levels of MDA. It has been reported that *R. officinalis* L. can eliminate peroxyl radicals and inhibit the formation of hydroxyl radicals [123].

## Stress relief in situation of real danger

### Background

In the face of imminent or fanciful danger, the organism precedes these events and intensifies some chemical reactions, generating a series of physiological signals that may affect the senses and some systems and cause symptoms, such as tachycardia, intense phobia, excessive perspiration, abdominal pain, and autonomic nervous system dysfunction [127]. Thus, the anxiety is installed. For the treatment, anxiolytics and antidepressants medications have been used, including the benzodiazepines, which are highly addictive and therefore should be consciously consumed [128]. However, many of these medicines may cause side effects such as hypotension, arrhythmias, and anticholinergic effects [129]. Selective serotonin reuptake inhibitors (SSRIs) are medications used to treat anxiety, being the most commonly prescribed antidepressants. Nevertheless, this drug may present side effects, including nausea, vomiting, insomnia, restlessness and dysfunction [130]. Due to these undesirable results, the use of plant medicines as adjuvant or primary treatment has been considered to control psychiatric, and neurological disorders [131].

### Methodology

**Fig. 2:**
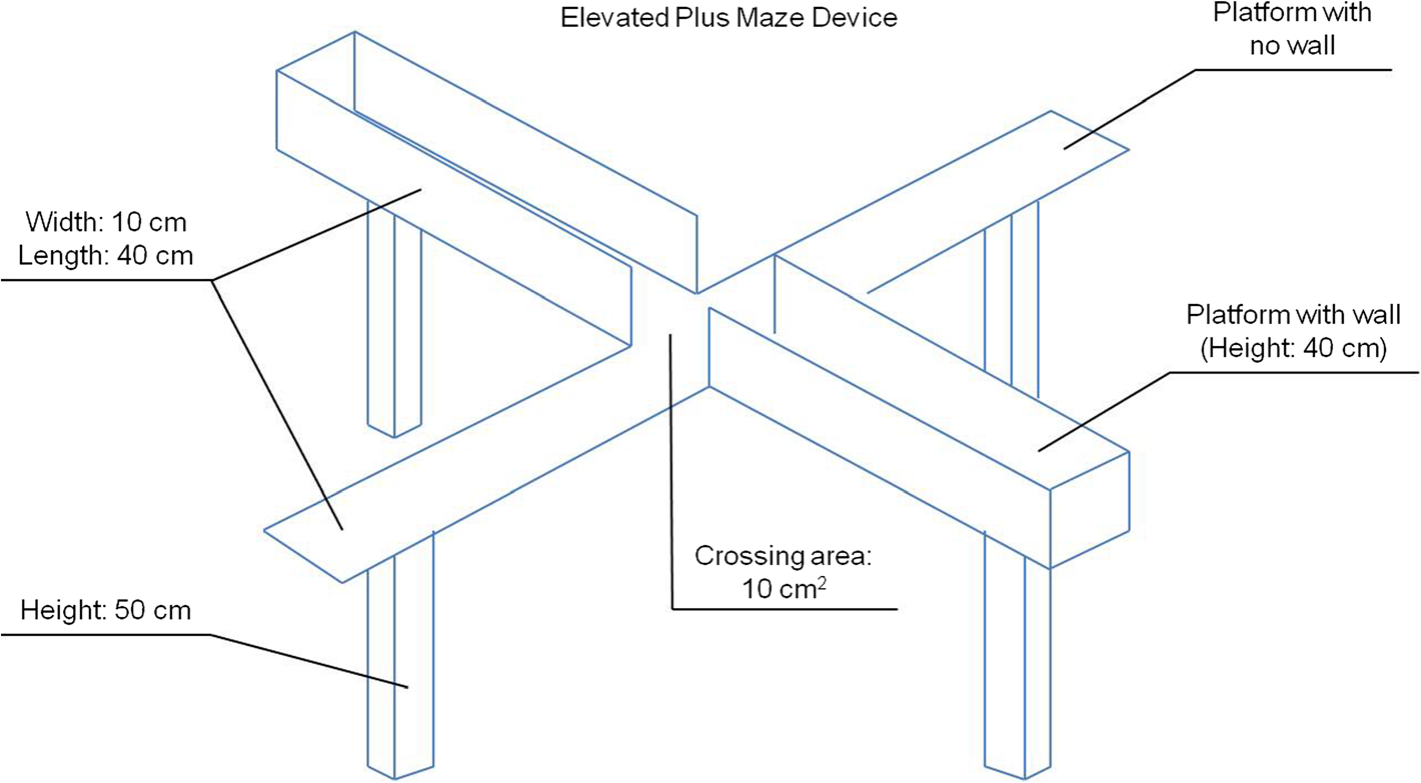
Elevated plus maze device, used to generate and measure the anxiety, as well as to check the effect of anxiolytic medicines. It is composed of two platforms that cross each other. One of them is walled, while the other has no wall. The center of the labyrinth has an area of 10 cm^2^, where the animal is placed with its head facing the non-walled region. The test is conducted for 5 min. Illustration based on real device

The antianxiety effect of *R. officinalis* L. hydroethanolic extract was evaluated in rats submitted to a stressful situation [132]. In this study, the animals received doses of plant extract (100, 200 or 400 mg/kg) by intraperitoneal injection. Rats from the control groups received saline or diazepam (1 mg/kg). The effect of the products on anxiety in rats was evaluated by the elevated plus maze device, which is used to generate and measure the anxiety, as well as to check the effect of anxiolytic medicines. It is a device composed of two platforms (width: 10 cm; length: 40 cm) that cross each other. One of the platforms is walled (high: 40 cm) while the other has no wall. The center of the labyrinth has an area of 10 cm^2^, where the animal is placed with its head facing the non-walled region. These platforms are 50 cm above the ground. An illustration of this device can be seen in Fig. 2. This test lasts for 5 min, and the animals are often stressed due to device height and unprotected regions; thus, they tend to seek shelter in walled areas. Hence, the anxiety may be measured by the entries and permanence of the animal in the protection-free area. Consequently, the absence of anxiety is related to the ability to cope with these challenges. The permanence time and the entries number in each maze area are quantified separately. The time spent and the entries percentage in unprotected regions, as well as the locomotor activity, should also be evaluated to measure the anxiety of the animal. The entries number in any maze may measure locomotor activity. This device was used in the study by Abadi et al. [132], which rats were previously injected with the products according to their experimental group and the test was conducted after 45 min.

### Findings

The permanence of the animals treated with *R. officinalis* L. extract in the regions with no walls was increased, indicating that these rats were less anxious and stressed. In this evaluation, higher concentrations of the extract were more effective and the dose of 400 mg/kg provided a similar effect to the diazepam. Additionally, the permanence time in protected areas was reduced using the extract at 400 mg/kg, similar to the standard medicine. Regarding the entries number in protected areas, a significant reduction was observed according to the dose, while in areas with no walls the entries number was increased. By locomotor activity analysis, it was found that the animals of all the groups presented similar behavior regarding the exploration of the maze. Based on these results, the antianxiety potential of *R. officinalis* L. extract was effectively demonstrated. The capacity this plant product to provide stress relief in situations of real danger could be an alternative to the conventional medicines, which may lead to various side effects and addiction.

#### Action mechanisms

The anxiolytic effect of *R. officinalis* L. can be attributed to its potent antioxidant capacity. With this, the brain can be protected by the many active molecules of the plant against the damages caused by free radicals. Possible routes of action include oxidative stress reduction and apoptosis inhibition that result in serotonergic neurons protection and anxiety reduction. Besides the antioxidant properties, *R. officinalis* L. has a significant anti-inflammatory effect. Thus, the plant contributes to reduce the inflammatory mediator’s levels, control the protein denaturation, and decrease the dopaminergic and serotonergic neuronal damages [132].

Anxiety has been treated with benzodiazepines, as diazepam. Using interaction with brain receptors for GABA neurotransmitter, diazepam can provide the anxiolytic effect and also act as a sedative [133]. In the study by Abadi et al. [132], administration of high doses of *R. officinalis* L. hydroalcoholic extract provided a similar effect to this drug.

The anxiolytic effect of *R. officinlais* L. can occur due to many phytocompounds in the plant. These molecules may act throughout the central nervous system [132].

It has been known that *R. officinlais* L. is very rich in flavonoids which work as ligands for central nervous system receptors [134]. One of these flavonoids, is the apigenin that can cross the blood-brain barrier and increase the effect of GABA neurotransmitter on its receptor in the neuron. This is an important inhibitory neurotransmitter of the central nervous system. Positively, the plant product does not cause dependence, as the use of benzodiazepines [135]. Another active flavonoid is the luteolin, capable of providing sedative and anxiolytic effects since it readily binds to the GABA receptors [136].

## Human tumor cells proliferation inhibition

### The nature of the tumors

Solid tumors consist of tumor cells and a characteristic tumor vascularization, which is different from the vascularization of healthy tissues. Thus, this microenvironment is physiologically formed by high interstitial fluid pressure (IFP), low oxygen tension, and low extracellular pH. The regular balance of growth factors present in healthy tissues is totally unregulated in a tumor tissue. This fact contributes to the development of an abnormal vascularization that compromises tissue structure and function. Therefore, the nutrition and excretion of tumor tissue products are compromised [137].

The high IFP in the tumor tissue is due to the vascular content accumulation caused by poor tumor vascularization [138]. Thereby, factors such as decreased blood vessel activity and lymphatic, osmotic pressure, and contractility of tumor stroma, cooperate for increased IFP [138, 139]. As a consequence, the flow of cells with antitumor activity and therapeutic substances are greatly impaired in tumor tissues [140]. It was verified a heterogeneous distribution of oxygenation in the tumor tissue since some portions receive low concentrations of oxygen and others are not attended, due to the insufficient vascularization [141]. As for the extracellular pH, it was observed that tumor tissues have acidic pH [142], due to the accumulation of lactate, produced by the glucose metabolism, that inside the tumor cell is found in high levels [143, 144].

### Therapeutic barriers to treat the cancer

Anticancer therapy can be compromised precisely by the three factors cited above: high IFP, low oxygen tension and low extracellular pH.

The high IFP in tumors compromises the delivery of the antitumor agent, mainly antibodies and other proteins, by decreasing the vascular flow, as well as its transport from the circulation to the tumor. Patients with lymphoma or melanoma have shown better results with chemotherapy when decreased IFP occurs during treatment [145].

Deficiencies in tissue oxygenation can inhibit the therapeutic effect of radiation, since oxygen is a potent radiosensitizer that contributes to tumor cell death [146]. Also, hypoxia has also been reported as a problem in the treatment with chemotherapeutic agents requiring oxygen for maximum efficiency, such as mephalan, bleomycin, and etoposide [147]. Besides, lack of oxygen compromises the cell division, thus, antiproliferative drugs lose their effectiveness on tumor cells [148].

Acid extracellular pH can impair the delivery of many chemotherapeutic agents [149]. The acidic condition in the tumor tissue can affect many drugs at the molecular level, preventing these agents from crossing the cell membrane [150]. Additionally, some therapeutic molecules are sequestered by acidic endosomes located inside the tumor cell [151].

### Breast adenocarcinoma (MCF-7) and cervical adenocarcinoma (HeLa) [91]

#### Methodology

*R. officinalis* L. glycolic extract was added on the cells previously cultured in microplates for 24 h. The analyses were performed after exposure to the extract in different concentrations (25, 50 and 100 mg/mL) for 5 min, using the following assays: (i) MTT [3- (4,5-dimethylthiazol-2-yl) -2,5-diphenyltetrazolium bromide], which measured the action of reductase enzymes in viable cells, by the MTT degradation and formazan formation; (ii) neutral red (NR), with the ability to impregnate lysosomes in viable cells; (iii) crystal violet (CV), that can stain the cellular genetic material; and (iv) genotoxicity, to verify the micronuclei (MN) frequency, using a fluorescence microscopy and DAPI dye that present affinity for the genetic material. *R. officinalis* L. extract decreased the viability of MCF-7 and HeLa, as evaluated by the MTT, NR and CV assays.

#### Findings

At 100 mg/mL, a significant low cell viability was noted by MTT, NR and CV. At 50 mg/mL, a reduction was confirmed by NR and CV. On the other hand, at 25 mg/mL, the cell viability was not significantly affected by neither assay. Therefore, the *R. officinalis* extract in higher concentrations interfered with the development of tumor cells. Regarding the genotoxicity, the tested concentrations induce no damage to the cellular genetic material, since the MN frequency was significantly lower (MCF-7) or similar to the control group (HeLa). This can suggest that the *R. officinalis* L. extract protected the cells against DNA damages. The damages could be more harmful to these cells since they already present alterations in their genetic material.

### Epithelial colorectal adenocarcinoma (CaCo-2) and histiocytic lymphoma cell line (U-937) [152]

#### Methodology

*R. officinalis* L. extract was obtained in ethyl acetate (EAE) and methanol (MEE) at 0, 5, 10, 15, 20 and 25 μg/mL. The effect of both extracts was evaluated on primary peripheral blood mononuclear cells (PBMC), a non-tumoral line, for comparative purposes. Firstly, the cells were cultured in microplates for 24 h, and then were exposed to the extract for 72 h, checking the cell viability every 24 h by Trypan blue exclusion test. Cell cycle and apoptosis were evaluated by flow cytometry. Early and late apoptosis were also measured by staining with propidium iodide DNA fluorochrome and annexin test.

#### Findings

Both extracts presented a dose-dependent anti-proliferative effect on CaCo-2 and U937. Besides, the extracts showed better performance on tumor cells than on PBMC, after 48 h, since IC_50_ was two and four-fold higher compared to U937 and CaCo-2, respectively. This fact demonstrated the selectivity of the extracts to act on tumor cells. By cell cycle analysis, an increased of cell percentage in S phase was observed. In contrast, a decreased cell population was noted in G1 and G2/M phases. The EAE extract kept the cells in the S phase (62%) longer, inhibiting their transition to G2/M phase. On the other hand, MEE extract provided a decrease of CaCo-2 population in G2/M phase. Regarding the apoptotic effect, CaCo-2 and U937 showed late apoptosis, 21.8 and 20.6%, respectively. These results can prove that *R. officinalis* L. extracts inhibited the proliferation of tumor cells.

### Esophageal squamous cell carcinoma (KYSE30) and gastric adenocarcinoma (AGS) [153]

#### Methodology

*R. officinalis* L aqueous extract was evaluated on adherent cells in microplates after exposure for 24, 48 and 72 h. The analyses were performed by (i) MTT test; (ii) NR assay; (iii) apoptosis with ethidium bromide/acridine orange (EB/AO), evaluated by fluorescence microscopy, which analyzed the condensed chromatin, apoptotic bodies and necrotic cells; and (iv) cell cycle analysis (interphase) by flow cytometry after DAPI staining.

#### Findings

*R. officinalis* L. extract affected the viability of KYSE30 and AGS after any exposure time. Regarding KYSE30, IC_50_ values were 600, 180, and 150 mg/mL, after 24, 48 and 72 h exposure, respectively, by MTT assay; and 860, 270, and 200 mg/mL, respectively, by NR assay. For AGS, IC_50_ values were remarkably lower, being 4.1, 1.8, and 1.3 mg/mL, respectively, by MTT assay; and 4.4, 2.1, and 1.1 mg/mL, respectively, by NR assay. Thus, the extract was more effective for the gastric adenocarcinoma lineage. Besides, the cells showed fragmentation and condensation of nucleus and chromatin, apoptotic bodies formation and increased apoptotic cells amount, proving that the plant extract induced the cell death. These findings are in accordance with cell cycle results, which demonstrated a higher percentage of cells in the G1 phase (above 60%). However, this percentage was below 30% in the S and G2/M phases, significant in cases of cancers due theses phases are related to the beginning and end of the DNA synthesis. Thereby, *R. officinalis* L. extract acted as an antiproliferative agent, interfering with the synthesis of defective genetic material.

### Lung carcinoma (A549) [154]

#### Methodology

The cells were treated with different concentrations (2.5, 5, 10, 25, 50, 100, 150 and 200 μg/mL) of *R. officinalis* L. extract for 72 h. The antiproliferative effect of these concentrations was verified by (i) crystal violet test; (ii) clonogenic assay, used to check the cell survival and ability to form colonies; (iii) immunoblotting, used to quantify proteins, such as PARP (related to apoptosis), Akt (related to cell proliferation), mTOR and p70S6K (both related to increased protein synthesis and cell survival).

#### Findings

*R. officinalis* L. extract presented IC_50_ of 15.9 μg/mL. Additionally, the extract at 2.5 μg/mL inhibited the colony formation (39.3 ± 3.1% of control) and at 10 μg/mL almost caused total elimination (1.2 ± 3.1% of control) as seen by clonogenic assay. This fact demonstrated the potential of the extract to control the stabilization and development of tumor cells, essential factors for the tumor growth in living beings. The PARP levels decreased to 50 μg/mL, thus the extract could contribute to improving the apoptosis process. Besides, *R. officinalis* L. extract inhibited the Akt phosphorylation, contributing to the non-activation of this protein that is related to proliferation and survival of A549 cells. In this way, the most effective concentrations were 25 μg/mL (57 ± 5.04% of control) and 50 μg/mL (36.1 ± 4.9% of control). The Akt levels also decreased at 25 μg/mL (49.8 ± 5.3% of control) and 50 μg/mL (32.4 ± 0.7% of control). Therefore, *R. officinalis* L. extract could interfere with the Akt signaling in these tumor cells. When Akt is activated, the signaling of mTOR and p70S6K may occur and result in increased protein synthesis, proliferation, and cell survival. However, the extract provided a significant inhibition of mTOR phosphorylation (53.3 ± 10.9% of control) and p70S6K (57.2 ± 14.8% of control) at 50 μg/mL. Low levels of mTOR (84.5 ± 2.5% of control) and p70S6K (83.3 ± 2.5% of control) were also observed. Based on these results, *R. officinalis* L. extract inhibited the A549 proliferation, interfering in some mechanisms related to colonization, proliferation, survival and apoptosis.

### Action mechanisms

Cancer cells can survive and develop tumors higher than non-tumor cells, even under chemo- and radiotherapy conditions. Among the strategies used by cells, the capacity to form new colonies is an important ability they present [154]. On the other hand, these authors proved that *R. officinalis* L. extract could inhibit the formation of new colonies of lung cancer cells (A549).

The tumor cells proliferation occurs on the oxidative stress influence, which exerts on the cells a force for them to survive to this adversity. Therefore, this situation activates the redox signaling and, consequently, activator protein (AP-1) and NF-κB are also activated. Subsequently, tumor suppressor genes inhibition can also be observed [155]. In contrast, the antitumor activity of *R. officinalis* L. has been attributed to the antioxidant effect that the plant presents, such as free radicals elimination and lipid peroxidation control [156, 157]. This fact has been proven on cancerous lineages such as MCF-7 and colorectal adenocarcinoma cells (HT-29) [158, 159].

The cytotoxicity of *R. officinalis* L. observed on tumor cells can be related to the interference in the cell cycle and also to apoptosis induction. *R. officinalis* L. methanolic and ethyl acetate extracts impaired distribution of U937 cell cycle in S phase, and provided a decrease in the G1 and G2/M phases. In addition, the methanolic fraction of the extract inhibited the CaCo-2 growth, with a decrease in the G2/M phase [152].

In response to the action of a therapeutic agent, the tumor cell can release ROS to provide the necessary oxidative stress for its proliferation, using the mitochondrial pathway [160, 161]. However, rosmanol, a phytocompound from *R. officinalis* L., caused apoptosis in colorectal adenocarcinoma cells (COLO 205), increasing the levels of apoptosis-inducing factor (AIF) and cytochrome c [162].

Other phytocompounds from *R. officinalis* L. have been cited as responsible for antiproliferative activity against cancer cells, as the ursolic acid that can act on the NF-κB pathway and provide NF-κB phosphorylation repressors inhibition. Thus, this phytocompound can attenuate the action of agents involved in oncogenesis, such as COX-2, MMP-9, cyclin D1, C-Jun, and C-fos. The antioxidant potential of ursolic acid has also been observed [153]. Besides, carnosol has shown a blocking effect on NF-κB [163], and carsonic acid has neutralized ROS and, consequently, protecting cell membranes against lipid peroxidation [153].

There is a class of proteins that after activation by cleavage acts on the DNA repair or even lead the cell to apoptosis, in case of impossible repair of the genetic material. These enzymes are called poly ADP ribose polymerase (PARP), and were activated by DNA breaking caused by ROS or other reactive species [164, 165]. In the study conducted by Moore et al. [154] increased PARP cleavage was observed in A549 cells by exposure to the *R. officinalis* L. extract, indicating an induction to the apoptosis of these cancer cells. These authors also proved that the plant extract can control the Akt phosphorylation, an important enzyme responsible for the regulation of metabolism, apoptosis, and cell proliferation. Blocking of this pathway (P13K/Akt) can result in improvements in the cancer treatment by chemo- or radiotherapeutic agents [166]. Besides, Moore et al. [154] have also shown that the extract can inhibit the activation of mTOR and p70S6K, mammalian targets of rapamycin that are cancer signaling proteins. Probably, this occurs due to the ability of *R. officinalis* L. extract in interfering with protein synthesis by DDIT4 gene induction, which is capable of inhibiting the synthesis of mTOR and p70S6k [167].

## Final considerations and conclusions

In this Review, some pharmacological effects of products from *R. officinalis* L. were shown. These effects were widely demonstrated on diverse types of disorders including (a) cardiac remodeling after myocardial infarction [47]; (b) body weight and dyslipidemia [60]; (c) cerebral ischemia [71]; (d) pain [77]; (e) infections [91]; (f) hepato-nephrotoxicity by lead [123]; (g) stress and anxiety [132]; and (h) tumor cells proliferation [91, 152–154]. Thus, with this study, it was possible to verify the benefits of *R. officinalis* L. on specific health problems that can affect many people around the world.

Supplementation with fresh leaves from *R. officinalis* L. provided better survival rates in infarcted animals, as well as improving diastolic function, cardiac muscle hypertrophy, and heart functions and morphology, compared to the animals receiving only conventional treatment. Thereby, it was demonstrated that the use of this plant could be a complementary therapy to the usual procedure of cardiac remodeling after infarction [47].

The use of some drugs, like antibiotics, can cause increased lipids and fats in the blood, known as dyslipidemia. Accumulation of these molecules in the vessels can promote cardiovascular and cerebrovascular diseases. However, administration of *R. officinalis* L. aqueous extract to the animals with gentamicin-induced dyslipidemia provided weight gain inhibition, caused by fat accumulation due to treatment with the antibiotic. Other positive aspects were the liver weight reduction of these animals and organ repair to the trauma generated by the medicament. Thus, the plant extract could be a strong candidate to control dyslipidemia and, consequently, reduce the chances of manifestations, such as heart attack, angina, and stroke, which have affected several individuals in all continents [60].

Stroke is precisely caused by disruption of blood flow in the brain, either by a clogging (ischemia) or by a ruptured vessel (hemorrhagic stroke). However, both cause irreversible damage to the affected region that will compromise the individual’s performance in some way. *R. officinalis* L. hydroethanolic extract has been shown a neuroprotective effect in animals with artificially induced cerebral ischemia and previously treated with the plant product, presenting reduced neurological deficit. In addition, the weight of the animals and their lipid indexes in the circulation were controlled by the use of *R. officinalis* L. extract. These findings demonstrated a prophylactic effect of *R. officinalis* L. on an essential clinical manifestation caused by disruption of blood circulation in the brain [71].

Some medications to treat pain can cause side effects, such as cardiovascular, renal, and gastric problems. Thus, the effectiveness of *R. officinalis* L. ethanol extract and its phytocompounds ursolic acid and oleanolic acid was observed in animals previously treated with the plant extracts, and with formalin-induced nociception. Plant products administrated in association with ketorolac improved the antinociceptive effect. Thus, this study showed that products from *R. officinalis* L. did not affect the efficiency of an allopathic drug, in contrast, they potentialized the pain control, in a complementary manner [77].

Another subject of relevance to the medical community is the emergence of opportunistic microorganisms and resistant to the available antimicrobial treatments. Because of this, *R. officinalis* L. glycolic extract was used in alternative to those therapeutic agents to control the development of mono- and polymicrobial biofilms. Thus, significant reductions of microbial communities were observed after the plant extract application. Therefore, the plant product could be a potential alternative therapeutic agent to eliminate microorganisms and, consequently, inhibit the development of infections that can culminate in a fatality [91].

Lead intoxication is a public health problem in many countries due to the aspects of subsistence of their communities. Several disorders can be reported, and the treatment can also be harmful in the same way as heavy metal intoxication. Therefore, *R. officinalis* L. ethanolic extract has been shown as a protector to the liver and kidneys against the toxic effect promoted by the Pb. Prolonged use of the extract has inhibited the degrading action of Pb, such as loss of body weight and weight reduction of liver and kidneys, blood cells reduction, increase in the circulation of markers related to the liver and kidney damage, and an increase of necrotic areas in these organs. In this way, the use of *R. officinalis* L. could be a way for these communities to prevent the harmful effects of Pb [123].

*R. officinalis* L. has also been tested for anxiety, as an alternative to the available antianxiety and antidepressants, which can present side effects, including hypotension, arrhythmias, and addiction. For this, animals were submitted to a stressful situation; however, they previously were treated with *R. officinalis* L. hydroethanolic extract. It was noticed a significant anxiety control similar to the diazepam. Many anxiolytic medications are useful, but cases of addiction have been reported. Thus, *R. officinalis* L. could be an alternative for these cases, since its prophylactic effect against anxiety has been proven [132].

Another malignancy that has advanced in all countries and caused the death of thousands of people is cancer. Regarding the therapy, many agents anticancer have been studied for the treatment of the disease, as antitumor and antiproliferative drugs. One of the problems involved would be the drugs reaching the target since the tumor microenvironment limits the diffusion of these drugs. Another considered aspect is the side effect of antitumor therapies. Therefore, the development of alternative methods less invasive with fewer side effects to the patients has been discussed. An example of this is the experiments with plant products, such as antioxidant molecules and extracts. Products from *R. officinalis* L. have been evaluated and demonstrated effective antiproliferative action on several types of tumor cells. These products have provided interference in the cell cycle, as well as promoting apoptosis, in order to inhibit the proliferation and colonization of malignant cells. Besides, these products have also been biocompatible for other cell types. Thus, the results have been promising and could be effective against the tumor cells, with preservation of the healthy cells and the minimum of damage for the organism of the patient [91, 152–154].

Therefore, many research groups around the world have been engaging in the development of alternative and biocompatible products to treat the most diverse physiological disorders that affect humans. Conventional medications are effective; however, they can offer several side effects, including severe morbidities. Phytotherapy medicines, those that are produced from plant products, such as phytocompounds, extracts, essential oils, and tinctures, have been used as alternative or complementary medicines, due to scientific evidence of their beneficial effects.

In this Review, reports on *R. officinalis* L. benefits were presented to show that a plant product may control physiological disorders similar to or superior to the usual medications. Another point to consider is the demonstration of new treatment forms and pharmacological strategies that could be developed to reach as many people as possible in all Continents.

## Abbreviations

5-HT1A: 5-hydroxytryptamine receptors
A549: Lung carcinoma
AGS: Gastric adenocarcinoma
AIF: Apoptosis-inducing factor
Akt: Protein related to cell proliferation
ALP: Alkaline phosphatase
ALT: Alanine aminotransferase
AP-1: Activator protein
AST: Aspartate transaminase
ATP: Adenosine triphosphate
BAL: Dimercaprol
Caco-2: Epithelial colorectal adenocarcinoma
CAT: Catalase
CFU/mL: Colony-forming units per milliliter
cGMP: Cyclic guanosine monophosphate
COLO 205: Colorectal adenocarcinoma cells
COX-2: Cyclooxygenase-2
CRE: Creatinine
CV: Crystal violet
DMSA: Mgnesium dimercaptosuccinic acid
EAE: Ethyl acetate
EB/AO: Ethidium bromide/acridine Orange
EDTA: Ethylenediaminetetraacetic acid
ERU: Urea
GABA: γ-aminobutyric acid
GLY: Glycogen
HDL-c: High density lipoprotein
HeLa: Cervical adenocarcinoma
HMG-CO: 3-hydroxy-3-methylglutaryl coenzyme A reductase
IC_50_: Half maximal inhibitory concentration
IFN-γ: Interferon gamma
IFP: Interstitial fluid pressure
IL-10: Interleukin 10
IL-1β: Interleukin 1 beta
IL-6: Interleukin 6
iNOS: Nitric oxide synthase
KYSE30: Esophageal squamous cell carcinoma
LDL-c: Low density lipoprotein
LIP: Lipid peroxidation
MCF-7: Breast adenocarcinoma
MDA: Malondialdehyde
MEE: Methanol
MMP: Matrix metalloproteinases
MN: Micronuclei
mTOR: Protein related to increased protein synthesis and cell survival
MTT: 3-(4,5-dimethylthiazol-2-yl)-2,5-diphenyltetrazolium bromide
NaCl: Sodium chloride
NADH: Nicotinamide adenine dinucleotide hydride
NF-κB: Nuclear factor kappa-light-chain-enhancer of activated B cells
NO: Nitric oxide
NR: Neutral red
Nrf2: Nuclear factor (erythroid-derived 2)-like 2
NSAIDs: Non-steroidal anti-inflammatory drugs
p70S6K: Protein related to increased protein synthesis and cell survival
PARP: Poly ADP ribose polymerase
Pb: Lead
PbA: Lead acetate
PBMC: Peripheral blood mononuclear cells
PCA: D-penicillamine
ROS: Reactive oxygen species
SOD: Superoxide dismutase
SSRIs: Selective serotonin reuptake inhibitors
TC: Total cholesterol
TG: Triglyceride
TIMP-1: Tissue inhibitor of metalloproteinases 1
TNF-α: Tumor necrosis factor alpha
TPA: Tissue plasminogen activator
TrpV: Transient receptor potential cation channel subfamily V member 1
TSP: Tissues protein
U-937: Histiocytic lymphoma cell line
